# Supplementary material for: The Systems Biology Research Tool: evolvable open-source software
Source: BMC Syst Biol. 2008 Jun 29;2:55. doi: 10.1186/1752-0509-2-55 (PMC2446383; doi:10.1186/1752-0509-2-55)
Supplement: Additional file 1 — SBRT Archive. An archive of the current version of the Systems Biology Research Tool. [file 1752-0509-2-55-S1.zip › sbrt-1.4.0/doc/developers_guide/api/sbrt/shell/text/LongFormat.html]

LongFormat


|  |  |  |
| --- | --- | --- |
| **Overview** \| **Package** \| **Class** \| **Use** \| **Tree** \| **Deprecated** \| **Index** \| **Help** | |  |
| **PREV CLASS**   **NEXT CLASS** | **FRAMES**    **NO FRAMES**     **All Classes** |  |
| SUMMARY: NESTED \| FIELD \| CONSTR \| METHOD | DETAIL: FIELD \| CONSTR \| METHOD |  |


---


## sbrt.shell.text Interface LongFormat

**All Superinterfaces:**: Format, Formatter<java.lang.Long>, Parser<java.lang.Long>, SimpleFormat<java.lang.Long>

**All Known Implementing Classes:**: LongFormatV1

---

``` public interface LongFormat extends SimpleFormat<java.lang.Long> ```

This interface is used to represent a format for longs.

**Author:**
:   This interface was written and documented by
    Jeremiah Wright while in the Wagner lab.

---

| **Method Summary** | |
| --- | --- |
| `java.lang.String` | `format(long i)`             Returns a formatted string representation of the provided long. |
| `java.lang.String` | `format(java.lang.Long i)`             Returns a formatted string representation of the provided long. |
| `java.lang.Long` | `parse(java.lang.String i)`             Parses the provided string and returns its corresponding long. |
| `long` | `parsePrimitive(java.lang.String i)`             Parses the provided string and returns its corresponding long. |

**Method Detail**

### format

```
java.lang.String format(java.lang.Long i)
```

:   Returns a formatted string representation of the
    provided long.

    :   **Specified by:**: `format` in interface `Formatter<java.lang.Long>`
    :   **Parameters:**: `i` - the long to be formatted. **Returns:**: a formatted string representation of the provided long.

---


### format

```
java.lang.String format(long i)
```

:   Returns a formatted string representation of the
    provided long.

    :   **Parameters:**: `i` - the long to be formatted. **Returns:**: a formatted string representation of the provided long.

---


### parse

```
java.lang.Long parse(java.lang.String i)
```

:   Parses the provided string and returns its
    corresponding long.

    :   **Specified by:**: `parse` in interface `Parser<java.lang.Long>`
    :   **Parameters:**: `i` - the string to be parsed. **Returns:**: the long corresponding to the provided string.

---


### parsePrimitive

```
long parsePrimitive(java.lang.String i)
```

:   Parses the provided string and returns its
    corresponding long.

    :   **Parameters:**: `i` - the string to be parsed. **Returns:**: the long corresponding to the provided string.


---


|  |  |  |
| --- | --- | --- |
| **Overview** \| **Package** \| **Class** \| **Use** \| **Tree** \| **Deprecated** \| **Index** \| **Help** | |  |
| **PREV CLASS**   **NEXT CLASS** | **FRAMES**    **NO FRAMES**     **All Classes** |  |
| SUMMARY: NESTED \| FIELD \| CONSTR \| METHOD | DETAIL: FIELD \| CONSTR \| METHOD |  |


---
